# The NeuroSense PremmieEd Parenting Educational Intervention (PremmieSense)—A Neuroprotective Intervention for Preterm Infant-Parent Dyads: Reported Using the TIDieR Framework

**DOI:** 10.3390/children13070907

**Published:** 2026-07-09

**Authors:** Welma Lubbe, Kirsten A. Donald, Jessica Botha

**Affiliations:** 1 Division of Developmental Paediatrics, Department of Paediatrics & Child Health, Red Cross War Memorial Children’s Hospital, Neuroscience Institute, University of Cape Town, Cape Town 7700, South Africa; kirsty.donald@uct.ac.za; 2 NuMIQ Research Focus Area, Faculty of Health Sciences, North-West University, Potchefstroom 2531, South Africa; jessica.botha@nwu.ac.za

**Keywords:** programme development, preterm infant, parent education interventions, programmes, neuroprotection, neural synchrony, TIDieR

## Abstract

**Highlights:**

**What are the main findings?**

- PremmieSense was designed as a neurodevelopmentally informed parenting education programme, combining a picture-based booklet with flexible, facilitator-led discussions.
- Its modular structure and multilingual materials allow delivery in resource-restricted NICU settings, where staff availability and length of stay are unpredictable.

**What are the implications of the main findings?**

- Pragmatic and adaptable designs are essential for NICU education programmes in low-resource contexts.
- Stakeholder involvement and linguistic accessibility are critical to ensure fidelity, usability, and equitable parent engagement.

**Abstract:**

Background: Preterm birth affects about 10% of births globally, often resulting in neurodevelopmental delays and disrupted parent-infant bonding. In low-resource settings, such as South Africa, neonatal intensive care unit (NICU) interventions require contextual adaptation. The NeuroSense PremmieEd Parenting Educational Intervention (PremmieSense) was developed to strengthen parent-infant bonding and promote neuroprotective care during NICU admission. Objectives: To describe the development, components, and pilot delivery of the PremmieSense intervention using the Template for Intervention Description and Replication (TIDieR) framework and document contextual adaptations and implementation lessons in low-resource NICUs. Approach: This project report outlines PremmieSense according to TIDieR. The programme comprises a picture-based booklet in English and Setswana, supplemented by facilitator-led group sessions delivered by trained healthcare professionals. It was piloted at two public-sector NICUs in the North West province of South Africa (*N* = 60 mothers; 30 per site). Parent knowledge was measured using the Knowledge of Preterm Infant Behavior (KPIB) scale, and stress was measured using the Parental Stress Scale: NICU (PSS:NICU tool). Quantitative outcomes are reported separately in a companion paper. Findings: PremmieSense was feasible and acceptable in low-resource NICUs. Logistical challenges including early discharges, staff constraints and language needs required pragmatic adaptations. The modular, multilingual design supported flexible delivery. TIDieR reporting facilitates replication and contextual adaptation. Conclusions and Recommendations: PremmieSense shows promise as a culturally appropriate and adaptable intervention for resource-constrained NICUs. Future work should tailor content to gestational age, prior parenting experience, and literacy, expand implementation, and assess long-term outcomes.

## 1. Introduction

### Rationale for the Programme

In 2020, preterm birth accounted for 9.9% of all births globally, with an estimated 13.4 million preterm births [1]. Southern Asia and sub-Saharan Africa account for 65% of all cases [1]. While in South Africa, the preterm birth rate was 13%, making it the country with the fifth-highest rate of preterm births [2]. These infants are at risk for short- and long-term adverse effects, including physiological challenges [3], medical conditions [4,5], sensory and behavioural challenges [3,6], and altered neurological and visual development [5,7,8].

The consequences of preterm birth extend beyond the infant. Parents frequently experience guilt, anxiety, loss and grief [3,9], anger and depression, hostility, and fear, in addition to high stress levels [10,11], and dysfunctional parenting [3,9]. Furthermore, parents may experience shock due to the potential critical condition of both the mother and/or baby, making preterm birth a highly stressful or even traumatic event for them [11,12], and an experience that has the potential to impair the relationship with their baby. In addition, infant-mother separation may lead to toxic stress in infants, with negative effects on both short- and long-term developmental outcomes [13].

In children born prematurely, the natural attachment process is interrupted at birth. When the mother and infant remain together without medical interference, close body contact regulates the newborn’s temperature, respiration, and energy conservation while simultaneously activating the mother’s caregiving responses, breastfeeding initiation, and hormonal regulation [14,15]. In preterm infants, this physiological process, which ordinarily unfolds in the supportive intrauterine environment and on the mother’s body, is interrupted. Instead, the immature newborn is exposed to the extrauterine environment of the NICU, where both the mother and infant are deprived of the normal processes that support development.

The NICU environment, while life-saving, can inadvertently contribute to emotional and physical separation within the parent-infant dyad, impairing early bonding and attachment [16]. Als’ Model of Synactive Organization of Behavioural Development [17] provides a theoretical foundation for understanding and addressing the disruption caused by preterm birth. The model explains how an infant transitions from the intrauterine to the extrauterine environment in preparation for functioning in the world [17]. This model underpins neuroprotective approaches to support preterm infants and informs the family-centred philosophy shared by established NICU intervention programmes. NICU interventions which make use of this model include the Newborn Individualized Developmental Care and Assessment Program (NIDCAP) [17], the Implementation of Neurodevelopmental Supportive Care Programme (INDeSC) [18,19], Wee Care Neuroprotective NICU Program [20], the Family and Infant Neurodevelopmental Education programme (FINE) [21], and the Infant- and Family-Centered Developmental Care (IFCDC) consensus standards [22]. While these programmes represent important advances in neuroprotective care, they are complex, multi-layered, and often organisation-wide in scope, making them difficult to implement in resource-constrained public-sector settings.

With effective interventions provided at critical times, the NICU also holds significant potential to support infant survival and development [23,24], promote parental mental health, and foster a healthy transition to parenthood [25,26,27]. Parent education, in particular, has demonstrated potential for enhancing parent–infant relationships, supporting positive behavioural development, promoting breastfeeding, and contributing to early neurodevelopmental and overall child growth [26,27]. Despite this, education programmes remain among the most poorly reported interventions in the NICU literature [28,29], limiting the understanding of their components, replicability, and impact.

In the South African context, the first author developed the Little Steps website in 2005 as an accessible, evidence-based resource to support parents through their NICU journey [30,31,32,33]. PremmieSense builds on the foundational work established by Little Steps [31,32,33] and is informed by a systematic review of parent education programmes in the NICU [34] and contextual input from mothers in the NICU [35]. The programme was developed to integrate current scientific evidence with contextual insights from the South African public sector.

This project report describes the development and pilot implementation of PremmieSense using the Template for Intervention Description and Replication (TIDieR) framework [36]. The TIDieR framework has been increasingly applied in health intervention reporting to support clarity, replication, and implementation, including in neonatal settings. For example, Tume et al. [37] used the TIDieR checklist to guide the development and description of a collaborative early intervention focused on parent–infant interaction in the NICU. TIDieR is an extension of the CONSORT 2010 statement (item 5) and SPIRIT 2013 statement (item 11) [36]. Applying TIDieR to PremmieSense provides a transparent and structured account of the intervention that will assist with its implementation, replication, and contextual adaptation in the future [38]. The quantitative pilot outcomes were reported separately [39].

## 2. Approach

### 2.1. Aim

This project report describes the PremmieSense programme in detail, using the TIDieR checklist [36], a 12-item framework designed to enhance the quality of intervention reporting and support replicability [38]. Feasibility and acceptability observations are noted where relevant to contextualise implementation decisions; formal outcome measures (parent stress and knowledge) are reported in the companion pilot study [39].

### 2.2. Programme Development

PremmieSense was developed for parents of preterm infants admitted to public-sector NICUs in South Africa, where resource constraints, staff limitations, and unpredictable lengths of stay present significant challenges for the delivery of structured parent education. The programme was designed to be pragmatic, modular, and linguistically accessible, addressing the gap between complex multi-component NICU programmes and the realities of low-resource clinical settings.

The content development team comprised a professional nurse, a research assistant, a mother with lived NICU experience, an academic expert, a master trainer of neurodevelopmental supportive care who was also a qualified speech-language pathologist and audiologist, a senior researcher in paediatric neuroscience, and an independent instructional designer. Programme content was informed by the systematic review findings [34] and direct input from mothers in the NICU [35], ensuring both evidence-based and contextually relevant content.

### 2.3. Pilot Setting and Participants

PremmieSense was piloted at two public-sector hospitals in the North West province of South Africa: a tertiary referral hospital (Site A) and a regional hospital (Site B). Site A is a high-acuity facility with an average NICU admission rate of approximately 66 infants per month, characterised by high patient turnover and short lengths of stay, often less than one week. Site B is a regional hospital serving a semi-urban population, where the social work department assumed the role of parent educators.

During the pilot, 183 mothers were recruited across the two participating hospitals (*n *= 104 at Site A and *n *= 79 at Site B) and allocated to one of three study arms: standard care (no booklet), booklet only, or booklet plus facilitator-led education. Sixty mothers (*n *= 30 per hospital; *n *= 10 per intervention arm) completed both the pre- and post-test assessments and comprised the analytic sample reported in the companion pilot paper [39]. Fathers were not included in the pilot study, as hospital practices at both sites discouraged their participation, a systemic limitation that is acknowledged and discussed further below. Inclusion was limited to mothers of preterm infants who were admitted to the NICU.

### 2.4. Outcome Measures

Parent knowledge of preterm infant behaviour was assessed using the Knowledge of Preterm Infant Behaviour (KPIB) scale [40], and parental stress was measured using the Parental Stress Scale: NICU (PSS:NICU) [41,42]. Both tools were administered as pre- and post-tests to participants in all three arms. The tools were administered at enrolment and again at least a week later, following a facilitator-led educational session. The KPIB and PSS:NICU were available in English; facilitators provided verbal translation and explanation where needed for Setswana-speaking participants. The quantitative outcomes of these measures are reported in full in the companion paper [39].

### 2.5. Reporting Framework

The programme is described using the TIDieR checklist as a reporting matrix, presented in numerical order in Table 1. The programme outline addresses each of the 12 TIDieR items and is described in detail in the Section 3 below.

## 3. Programme Description

The PremmieSense programme was initiated as soon as possible after NICU admission, recognising that early parental empowerment is critical to restoring the parent–infant relationship disrupted by preterm birth. The programme is described below using the TIDieR checklist [36] as a reporting matrix, with each item presented in numerical order, as summarised in Table 1.

### 3.1. Item 1—The Name of the Intervention

PremmieSense is a parenting educational intervention delivered to parents while their infants are admitted to the NICU. The programme is based on the principles of neuroprotective care and development. It aims to create awareness in parents regarding their preterm infants’ development and therefore empower them to fulfil their role as primary caregivers and provide sensitive, responsive parenting as early as possible during the NICU stay.

### 3.2. Item 2—The Rationale and Development

An understanding of the normal physiology of the newborn infant-parent dyad and how parent education can support or restore normal physiology provides the foundation for this programme. Physiological developmental processes refer to the ‘characteristics of or appropriate to an organism’s healthy and normal functioning’ [43]. These processes are observed in the mother–infant dyad when there is no medical interference and the dyad is kept together to ensure zero separation while initiating immediate and continuous skin-to-skin care. Winberg [14] explained that a mother who has close body contact with her baby helps to regulate a newborn’s temperature, energy conservation, respiration regulation, and acid-base balance, and improves breastfeeding. The baby also regulates the mother by increasing her attention to the newborn’s needs, breastfeeding initiation and maintenance, vagus nerve activation to ensure efficient energy use, and the release of gastrointestinal tract hormones to improve the use of ingested calories [14].

In preterm infants, this physiological development, which would be experienced in a supportive intrauterine environment and on the mother’s body, is interrupted. Immature newborns are exposed to an unsupportive extrauterine environment, where both the mother and baby are deprived of normal processes to support development. Researchers have demonstrated the negative effects of infant-mother separation, leading to toxic stress with negative effects on both short- and long-term developmental outcomes [9].

Evidence supports the value of restoring this connection as early as possible. Bergman [44] reported a 25% reduction in infant mortality when immediate and continuous skin-to-skin care was practiced. The effects of these changes can be detected months later. A notable twenty-year follow up study by Charpak et al. [45] compared kangaroo mother care (KMC) to traditional care. The findings demonstrate that KMC has lasting positive impacts on neurodevelopment, cognitive function, and social behaviour, underscoring the long-term benefits of early skin-to-skin contact.

Parental education is the first step in empowering parents to support the restoration of disrupted physiological processes. This educational intervention is informed by the theoretical mechanisms of neural coupling and synchrony, which are disrupted by preterm birth and the resulting separation between mother and infant, contributing to stress in both parent and child [46]. The programme seeks to reduce parental stress and enhance understanding of the infant’s needs. An important aim is to ensure access to information even in resource-restricted settings, creating awareness among parents about their preterm infants’ developmental activities and how they can contribute thereto.

The goals of the PremmieSense programme were formulated as follows:

- To enhance parent education, fostering stronger parent-infant relationships, promoting positive behaviour and temperament, supporting breastfeeding, and aiding early mental, neurodevelopmental, and overall child development.
- To prevent challenges that may disrupt parent-infant bonding and secure attachment.
- To empower parents with essential knowledge and skills to care for their preterm infants throughout their NICU stay.
- To build parental confidence in interacting with their infants, including recognising cues and providing responsive, supportive care.
- To create an optimal environment that facilitates neural coupling and synchrony between parents and their preterm infants, promoting healthy emotional and cognitive development.

### 3.3. Item 3—Informational Materials

The informational materials included in the PremmieSense parenting programme consist primarily of self-explanatory, picture-based, printed materials with concise text in either English or Setswana. Mothers were presented with a printed booklet to study on their own, followed by a face-to-face, lecture-based group discussion. For provider training, the lead researcher trained facilitators and provided them with a script to ensure consistent delivery. The materials were provided to facilitators fluent in local languages.

In resource-restricted settings, staff restrictions, and mothers being discharged or not available to visit every day make group-based lecture or bedside training challenging. A facilitator may not always be available to provide parental training, as care from medical and nursing staff is often triaged towards clinical care. By providing mothers with visual materials and explanations of what they could observe in their own infants and, when possible, augmenting the material with lecture-type group discussions, mothers were empowered to take on their parenting role, activating bonding and neural-coupling that had been interrupted due to separation and stress.

**Figure 1:**
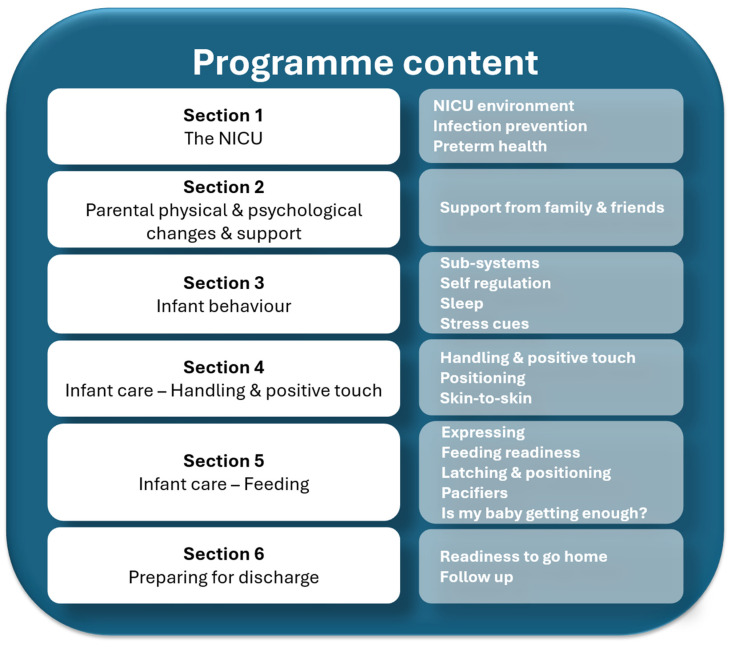
Programme content.

The printed booklets contain information about the NICU and preterm infants across six sections, identified through a systematic literature review [34] and direct input from mothers while in the NICU [35]. Figure 1 presents an overview of the programme content, while samples of the booklet and facilitator script are made available in the Supplementary Material.

The programme content was divided into six sections to allow for flexibility in delivery. Depending on the needs of the group, all or only selected modules could be presented, and sessions could be split and presented based on the availability of mothers and presenters. The modules of the programme include (see Figure 1):

Section 1—The NICU covers definitions of prematurity, how the intrauterine environment should be mimicked in the NICU, the importance of handwashing as part of infection control, and explanations of the equipment that parents may encounter around their babies.

Section 2—Parental physical and psychological changes and support provides short digestible support information which parents can consider and share with family and friends to build their support network while admitted to the NICU.

Section 3—Infant behaviour focuses on parent–infant interaction, covering the sub-systems of infant behaviour, calming behaviours, sleep and awake states, and stress cues parents may see in their infants.

Section 4—Infant care: handling and positive touch presents photographs to help parents identify calming strategies, illustrations of various positions suitable for preterm babies in the NICU, and information on skin-to-skin care.

Section 5—Feeding and breastfeeding were identified as the highest-priority topics by mothers. Since it can also be a more complex event, more detailed information is provided in this section. This section provides detailed information on breastmilk expression techniques, storage of expressed breastmilk, alternative feeding methods, the transition from tube to oral feeding, and assessing adequate milk intake Feeding is also the one activity which the mother–infant dyad can use to apply the knowledge gathered in the previous topics.

Section 6—Preparing for discharge addresses when discharge can be expected, feeding advancement post-discharge, and follow-up care. Although particularly relevant for parents of babies who are close to discharge, research supports beginning discharge preparation as soon as possible during the NICU stay. This section intentionally builds on the topics already included in the programme materials of previous sections.

### 3.4. Item 4—PremmieSense Procedure

Facilitators in each facility were identified and trained by the lead researcher on the programme content and provided a script to direct discussions. Facilitators were required to be knowledgeable about the NICU and neurodevelopmental care, fluent in mothers’ native languages, and available at times convenient for mothers.

The mothers received a printed booklet for independent self-study, followed by a facilitator-led, lecture-based group discussion. These structured, face-to-face sessions were held in small groups of two to four participants, allowing the facilitator to present materials, encourage questions, and guide discussion around topics that could be rearranged according to the needs of the group. This format allowed for interactive engagement and individual questions while remaining manageable within the constraints of the NICU setting. Facilitators were requested to provide one session (topic) per day, every day, or at least every second day, although in our context, this needed to be adapted (see item 10: modifications).

Although the programme includes topics such as infant behaviour, infant care, and feeding, hands-on demonstrations during caregiving or feeding times were intentionally excluded from the programme design. This was a deliberate decision to determine whether education alone, delivered through visual materials and structured group discussion, without accompanying practical demonstrations, would be sufficient to improve parental knowledge and confidence. In resource-restricted settings, facilitators are frequently unavailable during the times mothers interact with their infants, and consistent bedside support cannot be guaranteed. Focusing on knowledge transfer through accessible, self-directed materials ensures that the programme remains deliverable even when facilitated sessions cannot be arranged.

Ongoing quality assurance was ensured by reflection opportunities between facilitators and the lead researcher after group facilitation sessions to identify challenges and possible solutions. The effectiveness of the intervention was assessed by participants completing questionnaires before and after the sessions, measuring knowledge change (KPIB scale) and maternal stress (PSS: NICU) (see Lubbe and Donald [39] for quantitative outcomes).

### 3.5. Item 5—The Providers

At Site A, the programme was delivered by a professional nurse familiar with the NICU context and knowledgeable about the neurodevelopmental supportive-care model. She was trained in the content for this specific educational delivery by the lead researcher and provided with the same material that was given to the mothers, as well as text that could be used as a script to ensure consistent delivery across all parent groups. Importantly, the facilitator was fluent in English, Setswana and Afrikaans (the local languages of this region) and could therefore present the materials in all languages and facilitate discussions in the participants’ mother tongue.

At Site B, the hospital requested that social workers be trained as educators, as they frequently work with preterm infants’ parents. They were provided with the same training as those at Site A, spoke the three above-mentioned languages, and received the same training materials.

### 3.6. Item 6—The Delivery

The programme was delivered in person in the NICU between feeding times to allow mothers to attend and spend optimal time with their babies. Mothers were presented with a printed training booklet to study in their own time, followed by a face-to-face, lecture-based group discussion at the hospital within one week of enrolment. The booklets were handed to mothers in person by an independent fieldworker after the completion of informed consent and baseline questionnaires. In our pilot study, only two to four mothers participated at a time in lecture discussions, which allowed for good interaction and opportunities for individual questions.

### 3.7. Item 7—Where the PremmieSense Programme Was Delivered

The educational intervention was developed for the public healthcare sector and piloted in the NICU of a public tertiary referral hospital (Site A) and a regional hospital (Site B) in the North West province of South Africa. Mothers found it most convenient when the training was presented in a quiet area within the NICU, allowing them to remain close to their babies in case they were unexpectedly needed. The same delivery approach was followed at both sites.

### 3.8. Item 8—When and How Much

Mothers were enrolled in the programme and provided with a booklet (see Figure 1) as soon as possible following their infant’s admission, recognising that early support is critical during the initial and often traumatic period of NICU admission. Full details of the pilot design, including site descriptions, sample size, and allocation to study arms, are described in Section 2 above.

For mothers in the booklet and educational session arm, at least one group format, lecture-based session, was presented within one week of them receiving the booklet, at a time convenient to the mothers, which was usually in the morning between two feeds or early afternoon, when they were not at their infants’ bedside. Sessions lasted a maximum of 60 min each and focused on the needs identified by the group attendees. Mothers could attend any or all topic discussions (refer to Item 3, Figure 1) based on their individual needs and preferences.

### 3.9. Item 9—Tailoring of PremmieSense

Although the sessions were designed to follow the chronological development and experience in the NICU, the programme was dynamic in nature, and sessions could follow a different organisation and consist of any variety of topics or only a few selected topics, depending on the mothers’ needs. The facilitators spent more time on topics in which the participants had more questions. Therefore, the developed materials remained static, but tailoring was done on an individual basis and based on participant questions, augmenting the printed materials.

### 3.10. Item 10—Modifications of the PremmieSense Programme

The original programme design, informed by international literature in phase 1 of the study [34], recommended a minimum of three educational sessions lasting 60–120 min each, delivered by trained healthcare professionals who were permanent staff in the unit. However, consistent with the findings of Helmer et al. [47], the number of sessions was reduced prior to implementation due to limited bed availability and high patient turnover in the participating NICUs. In this context, infants are frequently discharged before reaching optimal gestational or developmental milestones to accommodate higher-acuity admissions, or may be moved to different wards or facilities in the event of an emergency such as surgery or infection. Despite consistently high admission and occupancy rates, the short length of stay, often less than a week, meant that mothers who were not lodging in the hospital or did not visit frequently were unable to attend the full series of sessions as intended.

All mothers recruited in Arm 3 (booklet + education session) of the pilot study received an educational booklet at their enrolment. Where a mother was discharged or transferred before the group education session was conducted, she did not receive the full intervention but retained her booklet. Where discharge or transfer occurred after the session but before the post-test assessment, the mother had received the intervention but was unable to complete the outcome measures. In both instances, these mothers were not included in the post-test data, and only mothers who completed both the pre- and post-test assessments were included in the outcome analyses (reported in Lubbe & Donald [39]).

A meta-analysis by Caporali et al. [48] found that parental stress in the NICU is largely independent of infant medical risk, with disruption of the parental role emerging as the strongest contributor to parental stress. Limited opportunities to participate in infant care and restrictions on caregiving negatively affect parents’ emotional and physical wellbeing, regardless of infant acuity. Therefore, providing all mothers with the parenting education booklet was considered appropriate to ensure equitable access to information and support, even when discharge or transfer prevented participation in the full intervention.

To address language accessibility, a Setswana-speaking professional nurse with NICU experience, affiliated with the unit but not part of the clinical staff, was engaged at Site A. While her familiarity with the unit supported continuity, her limited availability due to academic responsibilities introduced scheduling challenges, which were managed through flexible session timing. Mothers’ attendance was sometimes further limited by transport constraints and other caregiving responsibilities. Sessions were capped at 60 min to accommodate maternal fatigue and maximise engagement.

At Site B, the social work department assumed the role of parent educators, and their availability was more consistent, as determined by the patient load.

### 3.11. Item 11—Intervention Adherences of the PremmieSense Programme

At the start of the intervention programme, the design team discussed the content and programme structure to reach consensus, planning to present at least three facilitated, face-to-face, lecture-based, group discussion sessions from enrolment over a period of at least one week. The intervention was piloted and subsequently adapted, after which the design team reached a consensus on the final programme content and structure, ready for future rollout.

To maintain the integrity of the programme across all the mother groups and ensure fidelity and adherence, the following strategies were employed: detailed materials with scripts were developed to direct discussions; programme goals were clearly formulated; the lead researcher provided standardised training to the facilitators to deliver the educational intervention; and participant engagement and responsiveness were assessed using the KPIB and PSS:NICU tools as pre-and post-tests. Session observations were not conducted in this study; however, ongoing quality assurance was ensured by reflection opportunities between the facilitators and lead researcher after group facilitation sessions to identify challenges and possible solutions. The effectiveness of the programme was assessed by requesting participants to complete two questionnaires prior to and after the educational sessions (see Lubbe & Donald [39] for quantitative outcomes). The first determined knowledge change using the Knowledge of Preterm Infant Behaviour (KPIB) scale [40], and the second determined whether the programme had any effect on maternal stress using the Parental Stress Scale: NICU (PSS:NICU) [41].

### 3.12. Item 12—The Extent to Which PremmieSense Was Delivered as Planned

The aim was to deliver the PremmieSense intervention to all parents of preterm infants admitted to the NICU. However, despite the NICU admission rate of 66 infants per month (Site A), babies were often discharged in less than one week of NICU admission to make room for new, more critical admissions, often full-term infants who could not participate in the intervention in terms of the selection criteria. This short stay and facilitator availability impacted the feasibility of delivering a multi-session intervention and changed between the conception and delivery of the study. Facilitators adapted to present the entire booklet’s content in one 60 min or less session where they allowed mothers to ask questions and participate in discussion about the content.

During the study period, it became evident that the preterm infant rates decreased, probably as a result of employing healthcare workers in the community who encouraged pregnant women to attend antenatal care, which in turn contributed to a decrease in preterm births and admissions. This finding is aligned with the studies by Masten et al. [49] and Saaka and Sulley [50], which support the phenomenon that the work of community healthcare workers has a direct impact on preterm birth and NICU admission rates.

Although the initial plan was to train permanent NICU staff to deliver the sessions during quieter times, increased clinical workload made this unfeasible. As a result, external healthcare professionals were trained to facilitate the programme. To ensure language accessibility, a Setswana-speaking professional nurse with NICU experience was enlisted at Site A, although her availability was limited. This required coordination with the mothers’ schedules, which were often constrained by personal responsibilities. At Site B, the more consistent availability of the social work department, based on patient load, allowed for smoother session delivery. These adaptations highlight the importance of flexible programme design and the challenges of achieving universal delivery in a high-turnover public-sector NICU setting.

## 4. Discussion

Parent education in the NICU has been demonstrated to be an effective intervention for addressing the adverse effects of preterm birth on the parent-infant dyad [51,52]. Given the time and resource constraints faced by public sector hospitals in countries such as South Africa, it is crucial to implement a feasible, effective, and sustainable intervention programme. This project report describes the development and pilot delivery of PremmieSense, a parenting education intervention designed specifically for low-resource NICU settings, reported using the TIDieR framework to support transparency and replicability of the intervention.

### 4.1. How PremmieSense Differs from Existing Programmes

Several established NICU intervention programmes share the family-centred, neuroprotective philosophy that underpins PremmieSense, including NIDCAP, INDeSC, Wee Care, FINE, and the IFCDC Consensus standards. However, these programmes are complex, multi-layered, and typically organisation-wide in scope, requiring substantial institutional commitment, dedicated staffing, and resources that are rarely available in public-sector NICUs in low- and middle-income countries. They are also primarily designed for implementation in well-resourced settings, where staff training programmes, physical infrastructure, and consistent staffing ratios can be maintained.

PremmieSense was designed to address this gap. Rather than requiring systemic organisational change, it offers a modular, standalone parent education component that can be delivered flexibly within existing resource constraints. Its picture-based, printed booklet format allows for self-directed learning independent of facilitator availability, a critical feature in settings where staff are triaged towards clinical care and session delivery cannot always be guaranteed. The multilingual materials further distinguish PremmieSense from existing programmes, which have predominantly been developed in English-speaking, high-income contexts. PremmieSense was designed from the outset for linguistic and cultural accessibility in the South African public sector by providing materials in both English and Setswana and requiring facilitators fluent in local languages.

Additionally, while most existing programmes reviewed by Puthussery et al. [51] deliver parent education on an individual basis, PremmieSense uses a small group format of two to four participants. This allows for peer support and shared learning among mothers, adding a relational dimension to the educational experience that individual delivery cannot provide.

### 4.2. Feasibility and Contextual Adaptations

Preliminary piloting of PremmieSense suggests that the programme is feasible within this setting, although challenges such as facilitator availability highlight the need for further evaluation before sustainability can be confirmed. The quantitative outcomes of the pilot study are reported in a companion paper [39]. Furthermore, it can be delivered by healthcare providers with experience in the NICU and familiarity with neuroprotection and neurodevelopmental care. Training in neurodevelopmental care and standardised use of the PremmieSense booklet is needed, although formal certification to present such training is not required.

The programme is provided through a picture-based, printed booklet with concise text in the parent’s preferred language (English or Setswana in our study). This format is easy to deliver and is supplemented by face-to-face, lecture-based group discussions held at the hospital between feeding times. This approach was chosen because mothers in our previous study expressed a preference for lecture-based sessions. Additionally, since the mothers preferred the sessions to be facilitated in their mother tongue, the pool of available facilitators was limited to healthcare professionals who were both knowledgeable in neuroprotection and developmental care, familiar with the NICU environment, and fluent in the mother’s language of choice, which posed a logistical challenge at times.

Although several pragmatic adaptations were required during implementation, these did not alter the core components of the PremmieSense intervention. The essential elements of the programme remained the evidence-informed, picture-based educational booklet, its neuroprotective and family-centred content, delivery by trained facilitators using standardised materials, and opportunities for discussion and clarification. Adaptations were limited to implementation strategies, including the number and duration of facilitated sessions, the order and emphasis of topics, facilitator discipline, and session scheduling to accommodate contextual constraints.

### 4.3. Sustainability and Staffing

Reliance on external or non-permanent staff at both sites represents a significant challenge to long-term sustainability within the public health system. At Site A, the programme was delivered by a professional nurse engaged specifically for this purpose, whose availability was constrained by academic responsibilities. At Site B, social workers assumed the educator role, offering more consistent availability but representing a different professional profile than originally envisioned. These arrangements were pragmatic responses to real staffing constraints; however, they highlight the difficulty in embedding parent education into routine NICU care without dedicated resources.

Several strategies warrant consideration for sustainable integration. First, identifying and training a designated parent educator within the NICU, whether a nurse, social worker, or allied health professional, would reduce dependence on external facilitators and support continuity. Second, the self-directed booklet component of PremmieSense provides a valuable fallback when facilitator-led sessions cannot be delivered, and its role in the programme should not be underestimated. In our study, the nurse responsible for human milk bank coordination expressed interest in delivering parenting education sessions once the programme was fully rolled out, illustrating the importance of engaging all available stakeholders to identify the most suitable candidate for this role. Third, integrating PremmieSense delivery into existing staff workflows, for example, during quieter NICU periods or as part of routine parent engagement, may reduce the additional burden on clinical staff while maintaining programme fidelity.

### 4.4. Barriers and Facilitators

Several barriers to routine implementation emerged during the pilot study. High patient turnover and short lengths of stay meant that many mothers were discharged before completing the full programme, limiting exposure, particularly for mothers of moderate-to-late preterm infants despite their expressed support needs. Transportation constraints and other caregiving responsibilities further limited attendance. Facilitator availability, language requirements, and the need for scheduling flexibility all introduced logistical complexity.

Facilitators were identified as a key enabler of programme success. Where facilitators were consistently available, engaged, and fluent in mothers’ nativelanguages, sessions were well received, and mothers reported feeling supported. The small group format created a safe and relaxed environment for questions and peer support. The modular structure of the booklet allowed facilitators to respond to the specific needs of each group, supporting individualised engagement within a standardised framework.

A formal cost analysis of PremmieSense delivery has not yet been conducted and represents an important area for future work. Given the low-cost materials and relatively modest facilitator training requirements, however, the programme has the potential to be a cost-effective addition to NICU care in resource-constrained settings.

### 4.5. Inclusion of Fathers

Fathers were not included in this pilot study, as hospital practices at both sites discouraged their participation. This represents a systemic limitation that extends beyond the design of PremmieSense itself. The literature increasingly recognises the importance of paternal involvement in NICU care for optimal infant development and family functioning [53,54]. Excluding fathers not only limits the reach of the intervention but may also affect the longer-term sustainability of the parenting behaviours the programme seeks to promote, since fathers play a critical role in supporting mothers and in the infant’s broader caregiving environment.

Future iterations of PremmieSense should actively advocate for the inclusion of fathers. This requires engaging with hospital management and NICU leadership, as well as adapting programme materials and delivery to address the specific needs and perspectives of fathers. Broader systemic changes to protect and involve the family unit, including consideration of the physical NICU space to accommodate family participation, are needed to support more inclusive implementation [53,54].

It should be noted that pilot data [39] revealed that 88.3% of mothers who participated in PremmieSense were single at the time of their infant’s NICU admission. This suggests that even in the absence of institutional barriers to father participation, paternal involvement may be limited in this population. This finding should also be interpreted within the broader South African context, where caregiving frequently occurs within extended family networks, paternal absence is relatively common, and childcare responsibilities are often assumed by mothers and other female relatives because of historical, socioeconomic and cultural factors [55]. Accordingly, PremmieSense was designed to support the primary caregiver most likely to be present during the infant’s NICU admission. Whilst PremmieSense centres the mother as primary caregiver in this context, future iterations could explore extending the programme to alternative support figures. Grandmothers, aunts, and other family members may play a significant caregiving role for families of preterm infants [56], especially in low- and middle-income context and thus strengthen the sustainability of parenting behaviours beyond the NICU stay.

### 4.6. Strengths and Limitations

This new PremmieSense educational intervention focuses on providing educational support to parents of preterm infants admitted to the NICU in a resource-limited public-sector setting in South Africa. It was developed by a design team to consider various stakeholders, including bedside clinicians, academics, instructional designers, and, most importantly, the end-user, mothers of preterm infants in the NICU. It provides immediate, written, clear, and easy-to-understand material which can be consumed in a self-directed manner to make it easily accessible as soon as a baby is admitted to the NICU. The booklet can further be taken home, revisited, and shared with family members and other caregivers. The booklet is augmented by lecture-style group discussions, which provide an additional layer of peer support and a safe and relaxed environment where parents can engage with the material and ask questions, adding to the reflexive and individualised nature of the programme.

The programme’s primary limitation in the pilot [39] was the challenge of achieving universal delivery in a high-turnover public-sector NICU. A limitation of the lecture component is that it depends on the availability of healthcare professionals who are fluent in the most prominent local languages to present the lecture discussion sessions; as a result, it cannot be presented when needed but rather in a scheduled manner. Despite the aim to deliver lectures to all parents, the high turnover and early discharge of infants made it difficult for many parents to participate in group sessions, though they did receive the booklet. This indeed highlights a significant limitation in achieving universal delivery in a public sector NICU setting.

Although several strategies were implemented to promote intervention fidelity, including standardised facilitator training, the use of facilitator scripts, and post-session reflection with the lead researcher, fidelity was not formally evaluated using objective measures such as session observations, audio recordings, or fidelity checklists. Consequently, it cannot be confirmed that all intervention components were delivered consistently across facilitators and sites.

Due to the nature of the pilot study feasibility and acceptability were assessed through pragmatic means, including facilitator reflection sessions, informal maternal feedback and field notes, rather than through formal standardised measures. Future implementation projects should incorporate standardised measures such as participant satisfaction.

In addition, fathers were not included in this study, as hospital practices in the setting discouraged their participation. This represents a systemic limitation that requires attention in future interventions to support more inclusive family involvement.

## 5. Conclusions and Recommendations

This project report presents the PremmieSense programme, a neuroprotective, parent-centred educational intervention. Reported using the TIDieR framework, the programme description provides a transparent and detailed account of its development, components, and pilot delivery, enabling other researchers and clinicians to reproduce or contextually adapt PremmieSense for use in similar settings.

PremmieSense addresses a clear gap in the NICU parent education landscape in low-resource settings. Unlike existing multi-component NICU programmes that require substantial institutional infrastructure, PremmieSense offers a pragmatic, modular, and linguistically accessible approach that can be delivered flexibly within the constraints of public-sector NICUs. The picture-based booklet supports self-directed learning independent of facilitator availability, while facilitator-led group discussions add a relational and interactive dimension that mothers found valuable. Together, these components aim to restore the neural coupling and synchrony disrupted by preterm birth and separation, support early parent-infant bonding, and promote continuity of care from NICU to home.

Pilot implementation shows that PremmieSense is a feasible and acceptable education programme in low-resource NICU settings, although pragmatic adaptations were necessary in response to high patient turnover, short lengths of stay, staffing constraints, and language requirements. These adaptations did not compromise the integrity of the programme and instead highlighted the value of its flexible, modular design.

The following recommendations are made for the future development and implementation of PremmieSense:

- Facilitator sustainability: Future implementation should prioritise identifying and training a designated parent educator within the NICU, whether a nurse, social worker, or allied health professional, to reduce dependence on external facilitators and support consistent programme delivery.
- Content tailoring: Programme content should be stratified according to gestational age, maternal experience, and literacy levels to ensure meaningful engagement across diverse groups of mothers.
- Inclusion of fathers: Future iterations must actively advocate for the inclusion of fathers, engage with hospital leadership to address institutional barriers, and adapt materials to meet the specific needs of fathers and mothers.
- Broader populations: The programme should be extended to more diverse cultural, linguistic, and socioeconomic groups to strengthen its applicability across the South African public health sector and beyond.
- Longitudinal evaluation: Longer-term follow-up beyond the NICU stay is needed to assess whether knowledge gains and reductions in parental stress are sustained over time and translate into improved parenting outcomes in the home setting.
- Fidelity, feasibility and acceptability measures: Standardised measures of fidelity, feasibility and acceptability should be incorporated into future implementation.
- Cost analysis: A formal cost analysis of PremmieSense delivery should be conducted to support the case for scale-up in resource-constrained public health systems.

Together, these recommendations reflect the importance of responsive, inclusive, and contextually grounded strategies for delivering parent education in the NICU, particularly within the constraints and realities of public-sector healthcare in middle-income countries.

## Figures and Tables

**Table 1:** TIDieR items and description of the NeuroSense PremmieEd Parenting Educational Intervention (PremmieSense).

Item	Content	Key Components of the Programme
1	Name	NeuroSense PremmieEd Parenting Educational Intervention (short name: PremmieSense)
2	Why (rationale)	− To enhance parent education, fostering stronger parent–infant relationships, promoting positive behaviour and temperament, supporting breastfeeding, and aiding early mental, neurodevelopmental, and overall child development. − To prevent challenges that may disrupt parent-infant bonding and secure attachment. − To empower parents with essential knowledge and skills to care for their preterm infants throughout the NICU stay. − To build parental confidence in interacting with their infants, including recognising cues and providing responsive, supportive care. − To create an optimal environment that facilitates neural coupling and synchrony between parents and their preterm infants, promoting healthy emotional and cognitive development.
3	What (material)	− Printed materials, augmented by short lectures.
4	What (procedures)	− Picture-based, colour printed material with concise text in English or Setswana. − Structured, lecture-type group discussions to supplement the print material.
5	Who provides	− Healthcare professionals skilled in the NICU context and knowledgeable in the neurodevelopmental supportive care model.
6	How (mode of delivery)	− Booklet provided for self-study. − Lecture-based, small group discussions with two to four participants.
7	Where	− Public hospital NICU or lecture room.
8	When and how much	− Booklet provided on enrolment. − Lecture-based sessions within one week of enrolment. − At least one session (maximum dependent on parent needs). − In the morning, between feeding times, or early in the afternoon. − Maximum length of 60 min per session.
9	Tailoring	− Number of sessions depends on the availability of mothers to attend and availability of healthcare providers to present the sessions. − Maternal needs guide the number of topics presented.
10	Modification	− Flexibility incorporated with consideration of individual family needs.
11	Intervention adherences	− Considerations on adherence of the provision of the PremmieSense programme, and a written summary.
12	How well (planned)	− Lead researcher trained the facilitators and provided them with a script to direct discussions. − Deviations occur when resources and parental needs change. − Opportunities for reflection between facilitators and lead researcher following sessions to identify challenges and solutions.

NICU = Neonatal Intensive Care Unit, TIDieR = Template for Intervention Description and Replication.

## Data Availability

The data presented in this study are available on reasonable request from the corresponding author. The intervention materials (e.g., programme content, handouts, and facilitator guidelines) are not publicly available due to copyright restrictions and ongoing programme development. A sample of the intervention materials is provided in the Supplementary Materials for reference.

## Supplementary Materials

The following supporting information can be downloaded at: https://www.mdpi.com/article/10.3390/children13070907/s1, Supplementary Material: Premmie Sense Parenting Education Programme and Facilitator Script Sample.

## Abbreviations

COPE	Creating Opportunities for Parent Empowerment
CP	Cues Programme
FINE	The Family and Infant Neurodevelopmental Education
H-HOPE	Hospital to Home
IFCDC Consensus	The Consensus Committee of the Standards, Competencies and Best Practices for Infant and Family-Centered Developmental Care in the Intensive Care Unit
INDeSC	Implementation of Neurodevelopmental Supportive Care Programme
KMC	Kangaroo Mother Care
KPIB	Knowledge of Preterm Infant Behaviour scale
M-MITP	Modified Mother Infant Transaction Programme
MITP	Mother-Infant Transaction Program
NICU	Neonatal Intensive Care Unit
NIDCAP	Newborn Individualized Developmental Care and Assessment Program
PBIP	Parent-Baby Interaction Programme
PPI	Preventative Psychotherapy Intervention
PSS:NICU	Parental Stress Scale: NICU
TIDieR	Template for Intervention Description and Replication
Wee Care	Wee Care Neuroprotective NICU Program
