# Supplementary material for: The NeuroSense PremmieEd Parenting Educational Intervention (PremmieSense)—A Neuroprotective Intervention for Preterm Infant-Parent Dyads: Reported Using the TIDieR Framework
Source: Children (Basel). 2026 Jul 9;13(7):907. doi: 10.3390/children13070907 (PMC13407223; doi:10.3390/children13070907)
Supplement: Supplementary file 1 [file children-13-00907-s001.zip › children-4361976-supplementary.pdf]

Supplementary Material

PremmieSense Parenting Education Programme and Facilitator Script Sample

Below is a sample of the parenting education programme content, printed in booklets, along with the facilitator script. The programme materials, including the educational booklet and facilitator script, remain the intellectual property of the first author. The sample materials shared here are intended to illustrate the nature and pedagogical approach of the intervention. The complete programme materials are available from the first author upon reason request.

<table>
<tr><th>Educational Booklet Content</th><th>Facilitator Script</th></tr>
<tr><td>Section 3: Infant behaviour
Page 14 “I am stressed”: Visual images depicting infant stress cues (e.g., facial expressions, body posture, hand signals)
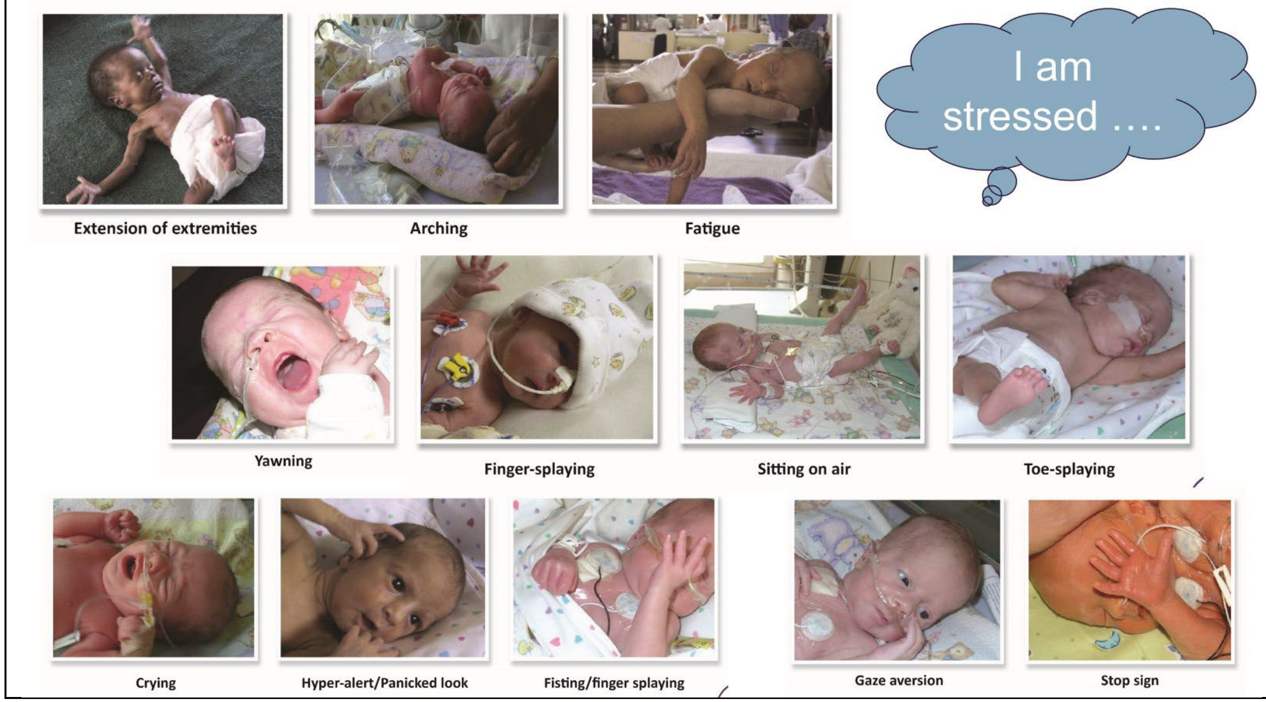
</td><td>Facilitator prompt/discussion question:
Have you seen these stress cues in your baby?

Facilitator note: Point to each stress cue depicted on the page and invite mothers to identify which cues they have observed in their own baby.</td></tr>
</table>

Section 1: The NICU, infection prevention, preterm health

Page 7: "Reading the monitors": Visual image of a NICU monitor displaying key vital signs: heart rate (HR), respiration, mean blood pressure (BP mean), and temperature.

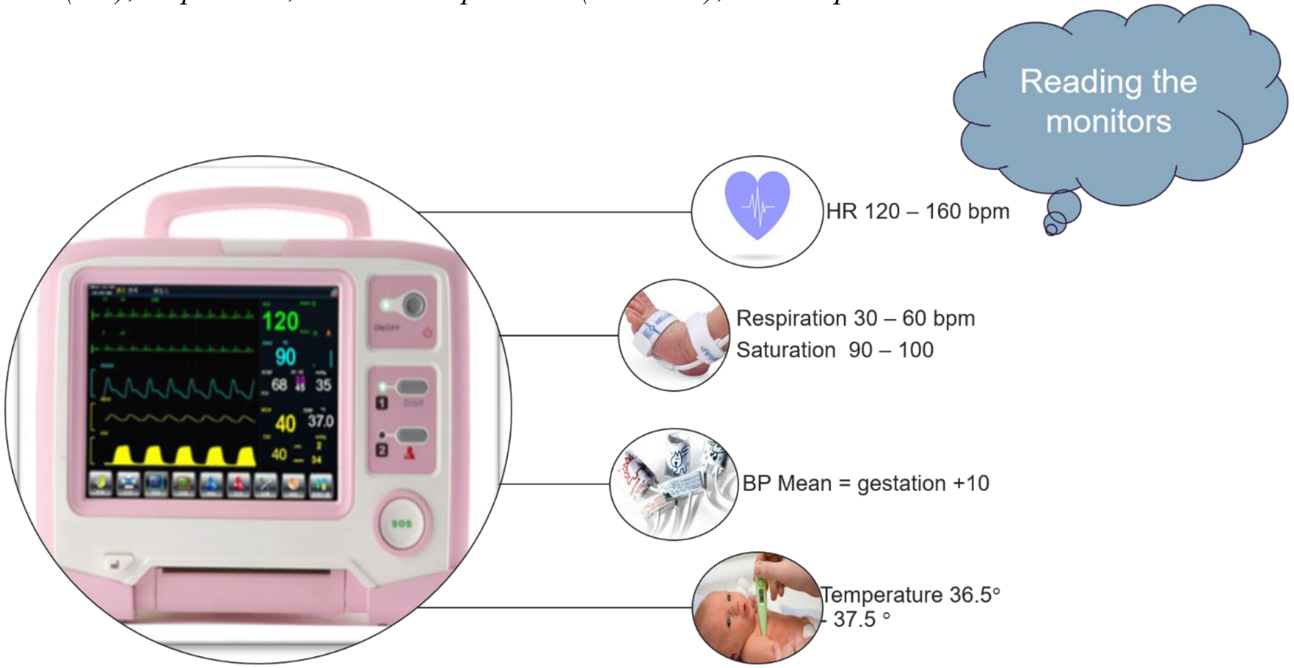

Facilitator prompt/discussion question: Looking at the monitors will tell you a lot about the baby's health.

Facilitator note: Guide mothers through each parameter shown on the monitor image, working from top to bottom. Explain that the top reading is the heart rate, shown as a spiky tracing, which should be between 120 and 160 beats per minute. The next reading shows oxygen saturation as a wave-like tracing, this should be above 92% in babies not receiving extra oxygen, or between 88–92% in babies who are. Note that oxygen levels that are too high can damage a preterm baby's eyes and cause blindness. Point out the blood pressure reading and explain that a rough guide is the baby's gestational age in weeks plus ten, and that stability matters more than the exact number. The bottom reading is the breathing rate, which should be between 30 and 60 breaths per minute. Explain that some preterm babies stop breathing due to immaturity, and that if this lasts 20 seconds or longer it is called apnoea. Finally, explain that temperature is critical; a cold baby uses extra energy, becomes ill, and can deteriorate quickly. The ideal range is 36.5°C to 37.5°C, and the best way to maintain warmth is skin-to-skin care on the mother's chest.

Section: Infant care: Part B (Feeding)

Page 25: "Latching my baby and breastfeeding positions" Sequential images illustrating the rooting, gape, and latch, followed by images demonstrating different breastfeeding positions.

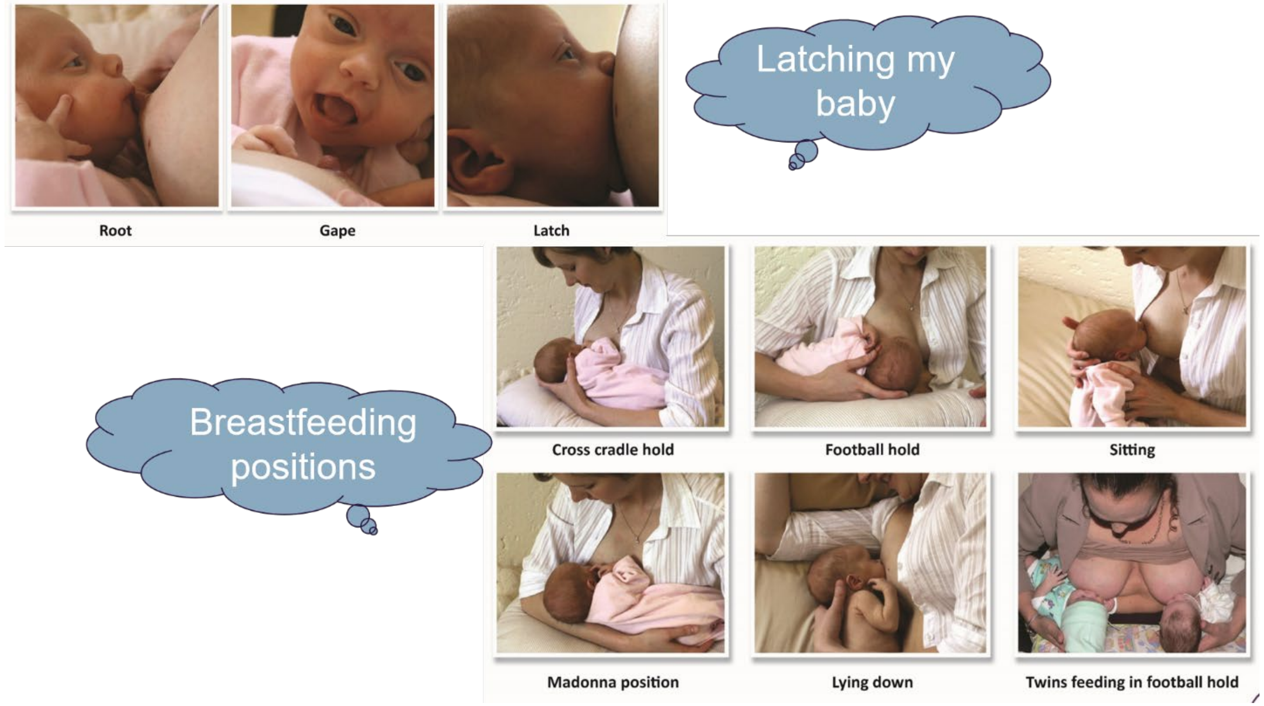

Facilitator prompt/discussion question:
Latching is very important to ensure good milk production and prevent sore and cracked nipples. There are different positions that you can try during breastfeeding and it is good to use different positions to ensure that the breasts are well drained to prevent blocked milk ducts.

Facilitator note: Guide mothers through the latching steps using the images: mother should sit comfortably with shoulders relaxed, holding baby with support at the neck and shoulders. Bring baby toward the breast and touch the mouth with the nipple. When baby opens wide like a yawn, calmly and quickly bring baby to the breast, allowing a large portion of the areola into the mouth. The nipple should point toward the baby's palate, and more of the dark areola should be visible below the bottom lip than above the top lip. Check positioning using the "tummy-to-mommy" guide: baby's tummy and mother's tummy should be parallel, with the ear, shoulder, and hip in a straight line and feet well supported. Then move to the position images and show each one.
